# Improving the teaching of “*correlation does not equal causation*” in Introductory Psychology

**DOI:** 10.3389/fpsyg.2025.1645518

**Published:** 2025-09-18

**Authors:** Courtney Stevens, Melissa R. Witkow, Jason Isbell

**Affiliations:** ^1^Department of Psychology, Willamette University, Salem, OR, United States; ^2^Department of Psychological Sciences, University of California – Merced, Merced, CA, United States

**Keywords:** pedagogy, introductory psychology, correlation, scientific reasoning, curriculum – undergrad and postgrad, curriculum & instruction, causal conclusions, critical thinking

## Abstract

*“Correlation does not equal causation”* is perhaps the most familiar phrase to any student or instructor in an Introductory Psychology class. While short and pithy, we argue that this phrase and its variants can lead to confusion among students, who may incorrectly interpret it to mean that *“correlation cannot mean causation.”* Unfortunately, this misinterpretation trades one type of reasoning error (incorrectly drawing a causal conclusion from a correlational study) for a separate type of reasoning error (incorrectly concluding there *cannot* be a causal relationship reflected in a correlational study). Drawing on patterns of student responses on an exam question targeting this issue, we demonstrate that this latter reasoning error is observed in over 30% of Introductory Psychology students. We end by proposing a set of possible sources of this confusion and call on instructors of Introductory Psychology to develop and assess methods to better teach this scientific reasoning skill.

## A common reasoning error

*“Correlation does not equal causation”* is perhaps the most familiar phrase to any student or instructor in an Introductory Psychology class. The phrase has several variants, including “*correlation does not imply causation,” “correlation is not causation,”* and “*correlation does not mean causation*.” All are shorthand ways of quickly communicating an important point about not drawing causal conclusions from correlational research designs, a common reasoning error made from correlational studies. In particular, the phrase can help students to understand that correlational designs provide weak evidence for cause-effect claims because there are alternative explanations that cannot be ruled out, such as reverse causality or causality attributed to a third variable. Many students quickly learn to repeat the familiar phrase when encountering examples of associations emerging from non-experimental research, and thus avoid incorrectly inferring causality when a variable was not experimentally manipulated. This can help students meet learning outcomes related to interpreting and evaluating psychological research identified by the American Psychological Association (2023). These scientific reasoning skills are foundational to the psychology curriculum, both for the major and the introductory course (American Psychological Association, 2021; Gurung et al., 2016; Mueller et al., 2020; Neufeld et al., 2022).

When teaching about research methods, instructors and textbooks often couple phrases like “*correlation does not equal causation”* with memorable examples, such as the association between ice cream consumption and shark attacks. The point of such examples is to demonstrate that even when two variables show a statistical association, causation cannot be definitively established without experimental manipulation of the variable believed to be causal. In the case of ice cream and shark attacks, because no variable was experimentally manipulated, there are three possible explanations for the association: ice cream consumption could cause shark attacks (A → B), shark attacks could cause ice cream consumption (B → A), or some third variable such as temperature could cause increases in both ice cream consumption and shark attacks (C → A and B). The ice cream / shark attack example is useful in supporting this instructional point precisely because of its absurdity relative to causal conclusions. Students can easily see why it’s unlikely that A causes B or that B causes A, and there is an obvious third variable (temperature) that can likely explain changes in both: as temperatures rise, people eat ice cream and flock to beaches where they can swim, but in doing so risk being attacked by sharks. This colorful example thus successfully reminds students not to draw a causal conclusion regarding a variable that has not been experimentally manipulated, i.e., in data emerging from a research design described as correlational or nonexperimental.

Prior work has tended to focus on the reasoning error described above, i.e., incorrectly inferring causality from correlational research designs (Bleske-Rechek et al., 2015; Hatfield et al., 2006; Xiong et al., 2019). This is an important focus given the suggestion by Matute et al. (2015) that illusions of causality, and a tendency to assume causality from coincidence, are human nature. For example, Hill et al. (2024) demonstrated differences in students’ causal attributions as a function of the variables involved, with little to no difference in causal attributions when the phrase ‘caused’ versus ‘associated with’ was used to describe the relationship. Other work indicates that particularly when the relationship between variables fits with expectations (e.g., a positive relationship between videogame playing and aggression), people are likely to infer a causal relationship, and importantly this is true *regardless* of whether the relationship is described as emerging from a study using a correlational or experimental design (Bleske-Rechek et al., 2015). As such, much instruction–and subsequent assessment–has focused on this reasoning error.

Past research has identified ways in which the language or data displays used to describe research findings can exacerbate the tendency to incorrectly draw causal conclusions from correlational designs. For example, Hatfield et al. (2006) focused on the possible confusion that occurs when students assume that the limitation to drawing causal conclusions is due to the *statistical* technique of correlation rather than the *methodological design* of nonexperimental studies (often referred to as correlational studies). Xiong et al. (2019) further explored how the type of data display can afford different reasoning patterns. Consistent with Hatfield et al. (2006), people are more likely to draw causal conclusions when nonexperimental data are displayed in a way that suggests a comparison between two groups (e.g., as a two-group bar graph). Xiong et al. (2019) speculate that this may be because this type of display is more consistent with an experimental than a correlational design.

## A second, less studied, reasoning error

Unfortunately, a focus on one type of reasoning error emerging from correlational designs may ignore a second reasoning error: incorrectly concluding there cannot be a causal relationship reflected in a correlational study. Yet, this latter error might be hidden if instructors only teach and assess students’ recognition that causal conclusions should not be drawn from a correlational study.

In Introductory Psychology, instructors often contrast correlational studies with experimental studies in which it may be possible to draw causal conclusions because alternative explanations can be ruled out. In particular, the randomized controlled trial (RCT) is often taught as a gold standard (or top of hierarchy) methodology that allows for researchers to appropriately draw a causal conclusion (for a discussion, see Wielkiewicz, 2024). At the same time, across many fields, there are complex discussions about the nuance and statistical procedures that may both limit conclusions from RCTs and allow stronger conclusions to be drawn from correlational data (e.g., see Deaton and Cartwright, 2018; Goldberg, 2019). According to Hill, who is credited with introducing the randomized controlled trial to medicine in the 1940s (Barrowman, 2014), the key question is whether “there is any other way of explaining the set of facts before us, is there any other answer equally, or more, likely than cause and effect?” (Hill, 1965, p. 299). A thorough discussion of these topics would be beyond the scope of Introductory Psychology, but it is important that students not get stuck with a hard and fast misunderstanding that a correlational finding means that a causal relationship is not possible. Thus, there are two reasoning errors to avoid making from a correlational study: (1) incorrectly drawing a causal conclusion from a single correlational study (the error that instructors commonly address through attention to reverse causality or third variable problems), and (2) incorrectly concluding a causal relationship is not *possible* among the relationships observed in a correlational study.

Both reasoning errors are important given the range and complexity of real-world research questions, some of which are not amenable to an RCT. This is particularly important in psychology, where many of the variables of interest (e.g., personality characteristics, exposure to environmental toxins, risk factors for depression) are unethical or impossible to manipulate experimentally. A correlation between variables is generally considered a necessary, but not sufficient, condition for establishing causality (but see also Barrowman, 2014; Deaton and Cartwright, 2018; Goldberg, 2019 for nuance on this point). Thus, it is critical that students recognize that associations in correlational research studies reflect *potential* causal associations. In other words, correlations between variables are one of many imperfect indicators that could support cause-effect relationships (Hill, 1965), particularly when placed in the context of a larger pattern of evidence (Wielkiewicz, 2024). Today, for example, we tend to accept that smoking is causally associated with lung cancer. However, Ronald Fisher, a smoker himself and consultant to tobacco firms, famously questioned whether the correlational evidence was strong enough to make the causal link between the two given the lack of an RCT (Fisher, 1957, 1958).

In an introductory course, students should know that while it is not possible to draw a causal conclusion from a single correlational study, the possibility of causation should not be ruled out. While the phrase *“correlation does not equal causation*” (or one of its variants) is commonly used to support students in learning not to make one type of reasoning error, it may fall short with respect to this second reasoning error. This may be especially likely when the phrase is coupled both with examples representing sets of variables that are obviously not causally related (e.g., ice cream and shark attacks) and instructional points focus on just the first reasoning error. The idea behind the phrase “*correlation does not equal causation”* could thus come to be misunderstood by students as “*correlation cannot mean causation.*” Unfortunately, this misinterpretation trades one type of reasoning error (incorrectly drawing a causal conclusion from a correlational study) for a separate type of reasoning error (incorrectly concluding there *cannot* be a causal relationship reflected in a correlational study). While less commonly studied, we argue this second type of reasoning error, or incorrectly concluding there cannot be a causal relationship reflected in a correlational study, is equally important. If students are making this second error, they may be ignoring valuable evidence or discarding *possible* causal relationships.

## Evidence of student errors

In our teaching at multiple types of institutions (community colleges, large-enrollment research universities, and small liberal arts institutions), we anecdotally observed many student comments in class that indicated the hidden error of incorrectly concluding that a causal relation was not possible in a correlational study. This suggested to us that some students may be making a reasoning error that we had not been addressing in our instruction or assessment. As a first step toward assessing students’ understanding of whether causal relationships can be established *or are possible* in a methodologically correlational study, we developed a multiple-choice question that could capture different types of reasoning errors. This single multiple-choice question was designed both to provide a first pass at quantifying our anecdotal observations of student reasoning errors and to stimulate thinking among instructors on ways to assess this type of reasoning error in their students.

**Figure 1:**
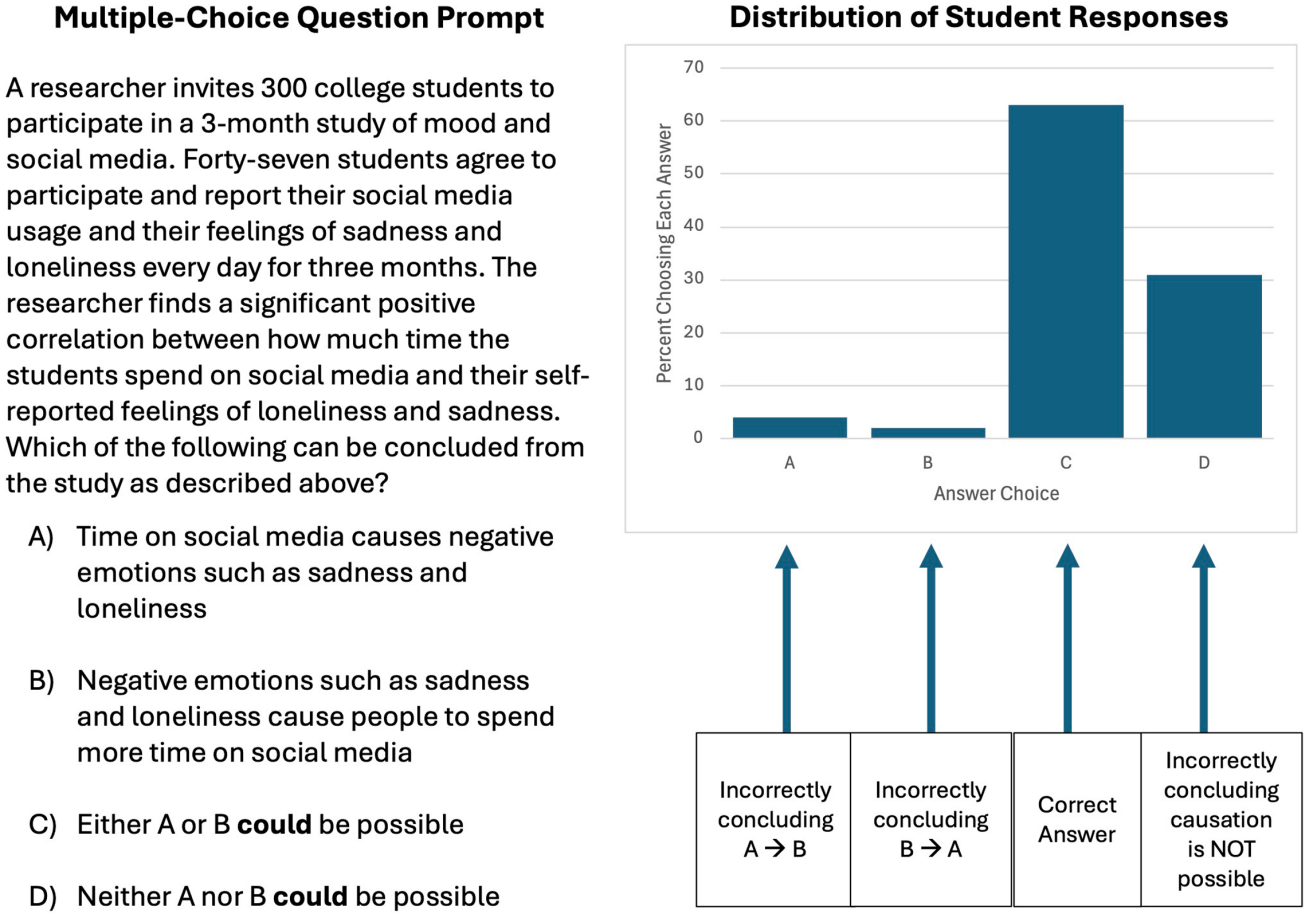
Multiple-choice question used to probe possible misconceptions in identifying what conclusions can correctly be drawn from a methodologically correlational study and distribution of student answer choices (*N* = 124 students). The correct answer is “C.” Incorrect answer choices reflect assuming causation in a particular direction (answer “A” or “B”) or thinking a causal relationship cannot be possible (answer “D”).

The question stem and answer choices are shown in Figure 1. The question stem presented a methodologically correlational scenario for variables that might be plausibly related in either direction (aspects of mood and social media use) and reported a significant positive correlation between the two variables. Based on the study as described, students selected which of four possible conclusions could be drawn. The correct answer required students to identify that it *could* be possible that there was a causal relationship in either direction. One incorrect lure assumed causality in one direction (A causing B) and another incorrect lure assumed causality in the other direction (B causing A). A student who had learned that a causal conclusion cannot be drawn from a correlational design might be expected to recognize these as inappropriate conclusions. However, an additional third incorrect lure indicated that a causal conclusion was *not possible* in either direction. This error could emerge if students have incorrectly come to believe that a causal relationship is not possible in a methodologically correlational design.

We administered this question on an exam to 124 students across five different sections of a single-semester Introductory Psychology taught by one of two instructors at a small four-year liberal arts college (the first and second author). All sections of the class were taught in-person. The assessment question was embedded in a unit exam (one of three for the course, worth ~20% of students’ final grade) that was administered proctored, in person, and closed-note / closed-book. Specific demographic information about students participating was not collected as part of this study, but typically two-thirds of students who take the Introductory Psychology course at this college are first year students. Within the undergraduate college, 99% of students are traditional college age (under age 26 years), and approximately two-thirds of students identify as mono-racial White. Both instructors, who each have taught Intro Psych at least 20 times, regularly use the phrase “*correlation does not equal causation*” as part of their teaching of research methods. Both instructors also talk about the three possible explanations for the observed relationship in a methodologically correlational study: namely that A could cause B, B could cause A, or some unknown third variable C could cause both A and B to change. Both instructors also provide examples to illustrate these possibilities, including at least one example in which the variables were clearly not causally related (e.g., ice cream and shark attacks).

Figure 1 presents the distribution of student responses across the four answer choices (one student did not make a selection and is not represented in the graph). The majority of students (63%) correctly identified that given the study design, it was *possible* that a causal relationship existed in either direction. Very few students incorrectly drew a causal conclusion in either direction (4% selected A causing B, and 2% selected B causing A), suggesting that students knew not to draw a causal conclusion from a methodologically correlational study. However, most notably, nearly a third of students (31%) incorrectly selected the answer choice indicating that a causal relationship was *not possible* in either direction. This reflects a “hidden error” that instructors may not be aware students are making if they are not assessing for it.

The distributions observed in the aggregate above were remarkably consistent across the five sections. Table 1 shows the distribution of answer choices by section, with 54–69% in each section selecting the correct answer but 23–46% incorrectly indicating that a causal conclusion was not possible in either direction. These data are drawn from students at a small liberal arts college, so it is unknown whether these rates are similar in other settings including community colleges and large universities, where less pedagogical research is conducted (Stevens et al., 2020).

**Table 1:** Distribution of answer choices overall and separated by class section.

Section	Chose A	Chose B	Chose C	Chose D	Total *N*
	*N* (%)	*N* (%)	*N* (%)	*N* (%)	
Section 1	2 (8%)	1 (4%)	15 (63%)	6 (25%)	24
Section 2	1 (4%)	0 (0%)	18 (69%)	7 (27%)	26
Section 3	2 (4%)	0 (0%)	18 (60%)	6 (23%)	26
Section 4	0 (0%)	1 (4%)	14 (58%)	8 (33%)	23
Section 5	0 (0%)	0 (0%)	13 (54%)	11 (46%)	24
Total	5 (4%)	2 (2%)	78 (63%)	38 (31%)	123

One student did not select any answer and is not represented in the table.

## Possible sources of confusion

Given that students incorrectly conclude that no causal relationship can exist in a correlational study at relatively high rates, why might that be? We propose several possible sources of this confusion below and hope that future work isolates these to identify their possible contribution to students’ reasoning errors.

One source of confusion might be the phrase “*correlation does not equal causation*” (or its variants) itself. In pedagogy, pithy phrases have utility and thus an understandable appeal. In being catchy, such phrases can facilitate recall. However, precision can be lost when using short catch phrases, and the resulting ambiguity can create space for misunderstandings. *“Correlation does not mean causation,”* if not read and considered carefully, might be readily misinterpreted as, “*correlation means no causation*.”

In other instantiations of the phrase, such as “*correlation does not imply causation”* precision itself could be the problem. In formal logic, the term “imply” indicates something is a sufficient condition, which has a technical definition: A condition which, if true, guarantees that a result is also true (Whitehead and Russell, 1910). In this case then, the phrase is definitionally correct. Correlation is not a sufficient condition that guarantees causation. In colloquial use, however, the word “imply” generally means to suggest a possibility. Interpreted in this way, the phrase suggests that when finding a correlation there is no need to consider the possibility of causation. Yet another version is, “*correlation does not indicate causation*,” which seems to preclude the possibility of causation altogether. Of course, the variability in the way this phrase is communicated (and explicated) across textbooks, instructors, or other teaching software might itself be a source of confusion.

A second source of confusion could be the types of examples given during instruction, which might have a biasing effect as well. As noted earlier, instructors often use memorable examples such as the association between ice cream consumption and shark attacks. These examples are often selected to address the concern that students might mistakenly assume correlated variables must be causally related. The heightened focus on these kinds of examples, especially if used to the exclusion of examples in which correlated variables are in fact causally related, might directly or implicitly bias students toward assuming that a correlation cannot mean causation.

Together, these potential sources of confusion point to an overarching issue in which Introductory Psychology courses may focus on one type of reasoning error from correlational designs to the potential exclusion of the other. Balancing course coverage to emphasize *both* types of reasoning errors might aid students in developing critical thinking skills and the ability to consider strengths and weaknesses of different research methodologies for drawing causal conclusions (Stevens et al., 2016; Wielkiewicz, 2024). In an era of big data and frequent “scientific” claims in social media (Barrowman, 2014; Matute et al., 2015; Wielkiewicz, 2024), this type of critical thinking and scientific reasoning is especially important (Mueller et al., 2020).

## Pedagogical implications

The phrase “*correlation does not equal causation”* (and its variants) can be effective at teaching students not to infer causality from a correlational study, but our experience in the classroom and the pattern of student responses on our single multiple-choice question suggest that many students incorrectly extend this to conclude that a causal relationship CANNOT exist. The comments we have heard students make in class and the pattern of errors on the multiple-choice assessment suggest that instruction can benefit from more precise wording and explanation, and we make several specific pedagogical suggestions below. While we approach these pedagogical issues from the perspective of Introductory Psychology instruction, the suggestions below span across the empirical sciences (Hill et al., 2024; Horton, 2023).

First, we recommend that instructors consider their choice of wording very carefully when using a short phrase to convey which types of conclusions can be drawn from a correlational study. For example, the use of “imply” in the phrase, “*correlation does not imply causation*,” readily lends itself to confusion given the notable difference between its technical definition in formal logic and its interpretation in colloquial usage. This may reflect confusion that arises from wording that may not carry its full intended meaning after being adopted into a different discipline or used in a different era. One alternative phrasing might be, “*correlation does not* guarantee *causation*.” Another option might be ‘*correlation does not* necessarily *mean causation*.’ In both examples, these small changes make the phrase longer but might more clearly communicate the possibility of causation. These revisions also suggest that more research must be done to establish a causal relationship, if the relationship exists at all. Of course, while catchy phrases can be useful tools in instruction, they are not requisite, and some instructors may wish to do away with the phrase and its variants altogether. As such, another option is to eliminate aphorisms and focus exclusively on the logic and application of correlation as a tool. More generally, instructors can be mindful to avoid causal and causal-inferred language when discussing correlational data, whether those data are presented as simple associations or in regression models. However, it is important to note that even this may have limited efficacy. Hill et al. (2024) showed that, for example, changing language from explicitly causal to “associational” did not seem to mitigate mistakes in causal interpretation among a sample of students.

However, this finding lends itself to a second recommendation, which is that instructors use a broad range of carefully considered examples when teaching about correlation and causality. A more even-handed presentation of examples, in which spurious correlations or third variable problems do not take center stage (as they do in the ice cream / shark attacks example), may help prevent students from assuming that “*correlation never means causation*.” One concrete example that might be pedagogically useful is the relationship between smoking and lung cancer, where a causal relationship is now accepted even though the supporting data are correlational. Instructors could present the range of correlational data observed at different historical time points, and the types of questions or alternative explanations raised (Fisher, 1957, 1958). Many real-world issues that students will encounter in psychology, medicine, public health, and beyond will be of this nature, and it is important for students to recognize when causal relationships are possible, alongside recognizing the strengths and weaknesses of different methodologies for ruling out alternative explanations. This can support students in moving toward more sophisticated and nuanced thinking about the nature and role of converging evidence. One of the most useful types of examples could demonstrate how combining observational/correlational studies with randomized controlled experiments can clarify possible causal relationships. These could include both examples where the randomized controlled trial *supports* causality and well as cases where causality is not supported. As the teaching community begins to address both types of reasoning errors, examples in these different categories could be developed and shared broadly.

Finally, in addition to addressing pedagogy, instructors should attend to assessment. That is, not only should instructors ensure that students do not mistakenly assume that correlation always means causation, but instructors should also ensure that students do not assume correlation never means causation. Assessments designed to identify the former error might be missing the latter error, and there is no need to trade one mistake for another. Intentionally looking for this second misconception might be important, as it may be invisible even among students who learn to parrot back the phrase ‘*correlation does not equal causation.’* This is especially true when assessment questions only test for the possibility of incorrectly concluding causality. An instructor may observe that students are correct in not drawing causal conclusions from correlational studies, but may not realize that some students are assuming that causal associations are not possible.

## Limitations and future directions

While the focus of this paper was to highlight a ‘hidden error’ among students and spur discussion among the Psychology teaching community, there are also limitations to the data presented that future work can address. First, while the single multiple-choice item used here provides a window into correlational reasoning errors, future work could use a broader range of questions and question types to assess student understanding and correlational reasoning errors more broadly. For example, different types of scenario stems or relationships (both clearly not causal and plausibly causal) could challenge students to consider these possibilities more deeply in different applied contexts. Instructors may also wish to include short-answer questions that require students to identify and discuss all possible causal explanations in a particular correlational scenario, including an assessment or consideration of their relative likelihood.

There are also potential problems with the specific item used here which suggest possible targeted manipulations in future studies. In particular, option (D) in the current assessment item may have been confusing to some students. A clearer option (D) could be worded as ‘Neither A nor B is possible.’ As well, in the four-alternative, forced-choice question used here, students may have been able to use test-taking strategies unrelated to their actual understanding of correlation and causation to rule out both causal conclusions (answer choice A and B), recognizing that it would not be possible for just one of those options to be correct. While in some correlational studies, causality in one direction can be ruled out due to temporal ordering or other factors, in this example that was not possible. An alternative type of question getting at this learning outcome might include a causal option only in one direction to address this limitation.

Expanding the assessment questions and including targeted manipulations could clarify the nature and scope of this reasoning error among students, as well as suggest areas for instruction and pedagogical innovation. This points to a related limitation, which is that while we have identified a reasoning error and suggest possible sources of the confusion and instructional remedies, we do not yet have data showing that specific changes will reduce the error in students. It will be important for instructors both to assess whether their own students are making this reasoning error and, if so, what pedagogical strategies are effective at reducing it. As instructors engage this type of reasoning error, we hope a set of evidence-based curriculum materials that support students’ correlational reasoning can be developed and disseminated.

## Conclusion

We hope this paper spurs increased discussion, teaching ideas, and pedagogical research among psychology instructors related to correlational reasoning. Interpreting and evaluating psychological research is a key learning outcome identified by the APA (American Psychological Association, 2023). As such, as a psychology teaching community it is imperative that we ensure that students understand the limitations of correlational designs (i.e., that we cannot draw causal conclusions from single correlational findings). However, students also need to understand that causal relationships are *possible* in a correlational design. We suggest that many students continue to make this latter error and call on the psychology teaching community to develop and evaluate methods to address this issue so that we do not continue trading one error for another.

## Data Availability

The original contributions presented in the study are included in the article/Supplementary material, further inquiries can be directed to the corresponding author.
